# Dyke-Davidoff-Masson Syndrome as a Rare Cause of Cerebral Hemiatrophy: Insights From a Case Series

**DOI:** 10.7759/cureus.54494

**Published:** 2024-02-19

**Authors:** Praveen K Sharma, Afwaan Faizal, Ajay Lucas Rubben Prabhu, Iffath Misbah

**Affiliations:** 1 Radiodiagnosis, Saveetha Medical College and Hospital, Saveetha Institute of Medical and Technical Sciences (SIMATS), Chennai, IND

**Keywords:** dyke-davidoff-masson syndrome, magnetic resonance imaging, computed tomography, mastoid, paranasal sinuses, seizures, paresis

## Abstract

Dyke-Davidoff-Masson syndrome (DDMS) is an uncommon neurological condition marked by changes in the skeletal structure, cerebral hemiatrophy, and ventriculomegaly. Manifesting primarily in early life, DDMS presents with seizures, hemiplegia, facial asymmetry, and intellectual disabilities. There are congenital and acquired types of DDMS, with ischemia being the most common cause of the latter. Three cases are presented here to highlight the radiological and clinical characteristics of DDMS. The first case involves a 27-year-old male with generalized seizures and right-sided hemiparesis since childhood, along with developmental delays and facial asymmetry. The second case features a 20-year-old male with recurrent seizures and developmental delays. The third case involves a 25-year-old female with uncontrolled seizures and learning difficulties since childhood. The clinical and radiological characteristics of DDMS are demonstrated in all three cases, emphasizing the significance of early detection and differential diagnosis. Imaging techniques, such as computed tomography (CT) and magnetic resonance imaging (MRI), which demonstrate ipsilateral ventriculomegaly, brain atrophy, and associated bone abnormalities, are highly helpful in the diagnosis. Differential diagnoses include Sturge-Weber syndrome, linear nevus sebaceous syndrome (LNSS), Silver-Russell syndrome, Fishman syndrome, and Rasmussen encephalitis. Treatment aims at managing seizures and associated disabilities, with hemispherectomy considered for eligible cases. This case series underscores the significance of prompt diagnosis and multidisciplinary management in improving outcomes for individuals with DDMS.

## Introduction

Dyke-Davidoff-Masson syndrome (DDMS), initially documented in 1933 through pneumatoencephalography and skull radiography abnormalities in a group of nine individuals, presents as an uncommon form of cerebral hemiatrophy. While adults and adolescents can be affected, it predominantly manifests in childhood and is attributed to early-life brain damage, often traumatic or prenatal [1-3]. Clinical features encompass mental retardation, hemiparesis, facial asymmetry, and recurrent seizures, along with speech and learning impairments [4]. Both genders may develop DDMS, with left-sided involvement and a male predominance noted [5]. Diagnostic imaging via computed tomography (CT) and magnetic resonance imaging (MRI) reveals characteristic signs, including cerebral hemiatrophy, ipsilateral ventricular dilation, and compensatory calvarial thickening. Ipsilateral paranasal sinus hyperpneumatization may or may not be present. Despite varied presentations, the core challenge remains intractable seizures, often necessitating surgical intervention due to an insufficient response to medication therapy [6].

## Case presentation

Case presentation 1

A 27-year-old male with intellectual disability, right-sided hemiparesis, behavioral problems, and generalized tonic-clonic seizures presented to the neurology department. The history of right-sided hemiparesis has gradually increased, with the first episode of seizure at the age of six years. According to the mother, the seizures began with bilateral upper extremity stiffness, which was followed by bilateral lower extremity involvement and mouth foaming. The last seizure occurred eight hours before admission and is said to have lasted for two minutes. His mother said that he had a history of developmental delays involving language, fine motor, gross motor, and social skills. No family member or sibling had ever experienced a similar problem before. Examining the face revealed asymmetry. During a neurological assessment, 4/5 power was seen in the right upper and lower limbs. Standard laboratory investigations like liver and kidney function tests, serum electrolytes, and random blood sugar levels were within the normal range.

**Figure 1:**
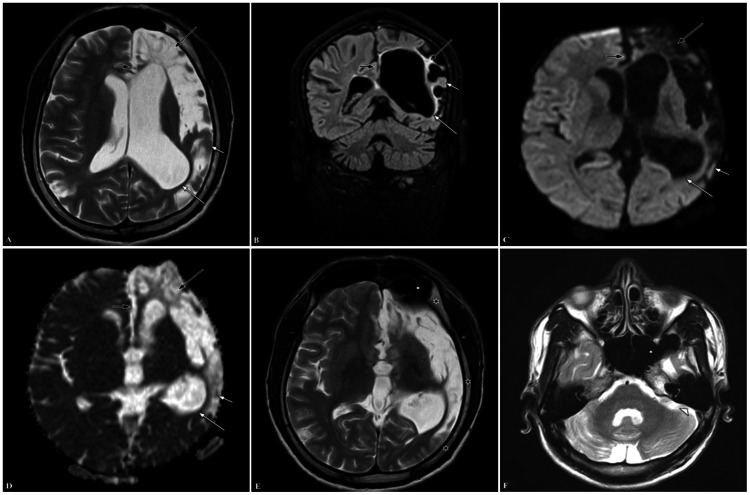
Case presentation 1 - MRI brain T2W axial (A), FLAIR coronal (B), DWI axial (C), and ADC axial (D) show left hemicerebral atrophy (short white arrow). There is a hyperintense signal on T2W, a hypointense signal on FLAIR, and a corresponding hypointense signal on DWI and a hyperintense signal on ADC in the cortical and subcortical white matter of the left cerebral hemisphere (predominantly in the left frontal lobe). A thin rim of a hyperintense signal on FLAIR representing gliosis (long black arrow) causing ipsilateral lateral ventricle ex vacuo dilatation (long white arrow) is indicated. There is a minimal midline shift of falx cerebri to the ipsilateral side (short black arrow). T2W axial (E, F) show compensatory ipsilateral calvarial hypertrophy (black asterisks), ipsilateral frontal and sphenoid sinus hyperpneumatization (white asterisks), and ipsilateral mastoid air cell hyperpneumatization (small white arrowhead), respectively. MRI: magnetic resonance imaging; T2W: T2-weighted; FLAIR: fluid attenuation inversion recovery; DWI: diffusion-weighted imaging; ADC: apparent diffusion coefficient

MRI study of the brain was done and showed left hemicerebral atrophy in the form of T2-weighted (T2W) hyperintensity, fluid attenuation inversion recovery (FLAIR) hypointensity, with corresponding diffusion-weighted imaging (DWI) showing hypointensity, and apparent diffusion coefficient (ADC) hyperintensity in the cortical and subcortical white matter of the left cerebral hemisphere (predominantly in the left frontal lobe). A thin rim of FLAIR hyperintense signal representing gliosis was seen with ipsilateral lateral ventricle ex vacuo dilatation and minimal midline shift of falx cerebri to the ipsilateral side. It was associated with compensatory ipsilateral calvarial hypertrophy, ipsilateral frontal and sphenoid sinuses, and ipsilateral mastoid air cell hyperpneumatization (Figure 1). The diagnosis of DDMS was made based on characteristic radiological findings and the patient was started on carbamazepine. Additionally, he underwent neurorehabilitation, including physiotherapy and speech therapy, to address functional deficits. Follow-up assessments demonstrated improved seizure control and motor function, with ongoing neurological follow-up and continued neurorehabilitation to optimize long-term outcomes.

Case presentation 2

A 20-year-old male with a poor socio-economic status came to the neurology department with an abrupt onset of involuntary movements of both limbs, eye gazing upward, mouth frothing, involuntary micturition, and tongue biting. When asked about his past by his parents, he had a normal, uneventful vaginal delivery, episodes of childhood-onset seizures controlled by medications, delayed achievement of milestones, and intellectual disability. Trauma or neuroinfection was not present in the past. There was no prior history of a comparable ailment among siblings or family members. He appeared moderately built, and an examination of his face revealed asymmetry and no cafe-au-lait spots. A neurological examination revealed unusual behavior along with disorientation to time and location. The results of routine laboratory testing, such as serum electrolytes and liver and renal function tests were within acceptable limits. An electroencephalogram (EEG) done elsewhere three years back revealed unusual EEG alterations, including diffuse background slowing and generalized seizure discharges.

**Figure 2:**
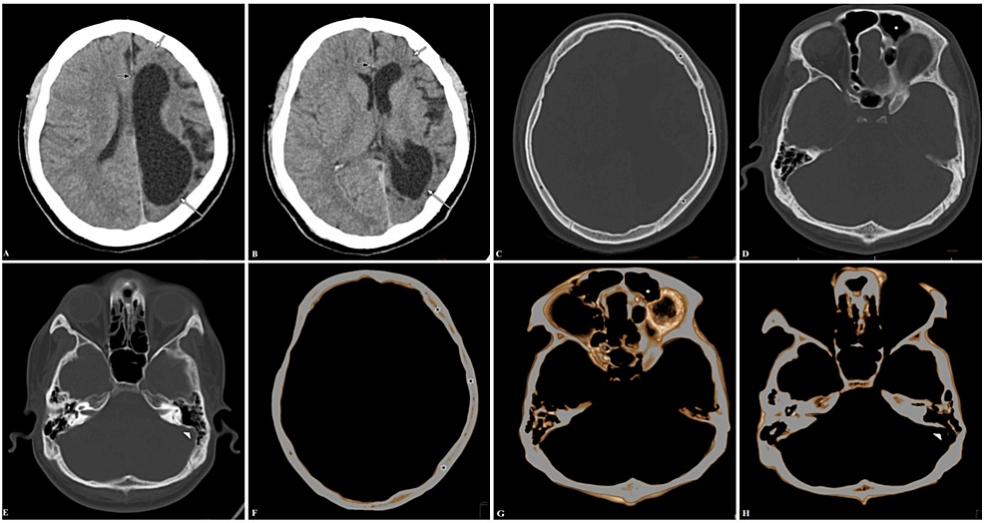
Case presentation 2 - CT brain Axial sections in the brain window (A, B) show left hemicerebral atrophy (short white arrow). There is ipsilateral lateral ventricle ex vacuo dilatation (long white arrow) and a minimal midline shift of falx cerebri to the ipsilateral side (short black arrow) at the corona radiata and Sylvian fissure level, respectively. Axial sections in the bone window (C, D, E) and 3D axial VR (F, G, H) show compensatory ipsilateral calvarial hypertrophy (black asterisks), ipsilateral frontal sinus hyperpneumatization (white asterisk), and ipsilateral mastoid air cells hyperpneumatization (small white arrowhead), respectively. CT: computed tomography; VR: volume rendering

CT study of the brain was done and showed left hemicerebral atrophy, causing ipsilateral lateral ventricle ex vacuo dilatation with minimal midline shift of the falx cerebri to the ipsilateral side. Other findings were compensatory ipsilateral calvarial hypertrophy, ipsilateral frontal sinus, and ipsilateral mastoid air cell hyperpneumatization (Figure 2). The diagnosis of DDMS was made based on the characteristic imaging findings and the patient was given two anti-epileptic medications upon discharge: phenytoin and valproic acid. In addition to pharmacotherapy, he underwent cognitive rehabilitation and vocational training to enhance adaptive skills and social integration. Subsequent follow-up revealed a reduction in seizure frequency and improved behavioral symptoms, necessitating continued antiepileptic therapy, neurorehabilitation, and psychosocial support.

Case presentation 3

A 25-year-old female arrived at the neurology department with uncontrolled seizure episodes each lasting at least three minutes, learning disabilities, slurred speech, and a history of medication-controlled childhood-onset seizures. The patient had no significant history of prenatal or postnatal issues and was born to non-consanguineous parents. According to her parents, she didn't have any vision or hearing issues. Examinations of the sensory system and cranial nerves revealed nothing abnormal. Neurocutaneous markers were absent. There was some mild facial asymmetry. Tests for liver, kidney, and blood were all within acceptable limits.

**Figure 3:**
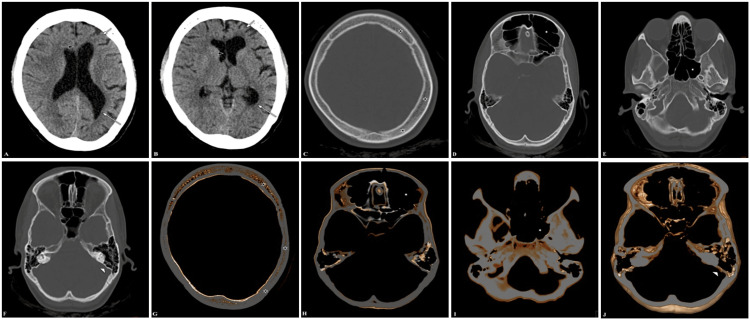
Case presentation 3 - CT brain Axial sections in brain window (A, B) show left hemicerebral atrophy (short white arrow). There is ipsilateral lateral ventricle ex vacuo dilatation (long white arrow) and a minimal midline shift of falx cerebri to the ipsilateral side (short black arrow) at the corona radiata and Sylvian fissure level, respectively. Axial sections in the bone window (C, D, E, F) and 3D axial VR (G, H, I, J) show compensatory ipsilateral calvarial hypertrophy (black asterisks), ipsilateral frontal and sphenoid sinus hyperpneumatization (white asterisk), and ipsilateral mastoid air cell hyperpneumatization (small white arrowhead), respectively. CT: computed tomography; VR: volume rendering

CT study of the brain was done and showed left hemicerebral atrophy, causing ex vacuo dilatation of the ipsilateral lateral ventricle with minimal midline shift of the falx cerebri to the ipsilateral side, compensatory ipsilateral calvarial hypertrophy with ipsilateral frontal and sphenoid sinuses, and ipsilateral mastoid air cell hyperpneumatization (Figure 3). The findings observed on CT, combined with the patient's clinical symptoms, met the criteria for DDMS. Following the initiation of carbamazepine, she participated in cognitive behavioral therapy and educational interventions to address cognitive and psychosocial challenges. Regular follow-up appointments indicated gradual improvement in seizure control, cognitive function, and overall quality of life, emphasizing the importance of sustained multidisciplinary care and long-term support.

## Discussion

The growing brain causes the bony skull table to expand outward, causing the child's head to grow to its maximum in the early years. By the end of the third year, the bony skull table grows to half its adult size. The essential sulci development is typically complete by the eighth month [7]. When the brain fails to mature normally, various structures begin to develop inward, leading to the enlargement of sinuses, elevated orbital roofs, and increased diploic gaps [8]. These alterations indicate damage occurring before age three and may manifest as early as nine months after the injury [9].

DDMS presents in two distinct variants. Congenital or infantile DDMS typically manifests during the perinatal period or early infancy and is attributed to fetal vascular occlusion, involving coarctation of the mid-aortic arch, precise anomalies in the middle cerebral artery territory, and unilateral cerebral arterial circulation [10, 11]. Conversely, acquired DDMS stems from diverse factors, including trauma, bleeding, ischemia, infection, hypoxia at birth, protracted febrile seizures, and tumors. Notably, ischemia stands out as the most prevalent cause, leading to reduced production of brain-derived neurotrophic factor and culminating in cerebral atrophy.

The typical clinical features depend on the extent of the brain injury. These manifestations include hemiplegia, convulsions, contralateral hemiparesis, and facial asymmetry. Additionally, individuals may exhibit cognitive impairments such as mental retardation, learning disabilities, and speech impairments. Seizures can be focal or generalized. A complex partial seizure with secondary generalization had also been reported [12].

The typical imaging findings on CT and MRI are cerebral hemiatrophy, ipsilateral ventriculomegaly, ipsilateral paranasal sinus hyperpneumatization, and compensatory ipsilateral calvarial thickening [13]. Associated findings include ipsilateral sulci enlargement, ipsilateral cisternal space dilatation, and reduction in the ipsilateral cranial fossa dimensions.

DDMS is differentiated from Sturge-Weber syndrome, linear nevus sebaceous syndrome (LNSS), Silver-Russell syndrome, Fishman syndrome, and Rasmussen encephalitis [14]. Encephalotrigeminal angiomatosis, commonly referred to as Sturge-Weber syndrome, is an infrequent condition distinguished by cerebral atrophy and a port-wine facial nevus. This syndrome is associated with leptomeningeal angioma, intracranial tram track calcifications, and the absence of midline shift [15]. LNSS, a rare disorder, is marked by distinctive facial nevi, mental retardation, recurrent seizures, and cerebral hemiatrophy accompanied by unilateral ventricular dilatation [16]. Silver-Russell syndrome, also uncommon, is characterized by stunted growth, delayed bone age, clinodactyly, average head circumference, normal intelligence, and a distinctive facial appearance featuring a triangular face, broad forehead, small pointed chin, and a thin, wide 'shark-like' mouth, along with hemihypertrophy [17]. Fishman syndrome, a rare neurocutaneous syndrome, manifests with features such as unilateral cranial lipoma, lipodermoid of the eye, seizures, calcified cortex, and hemiatrophy [18]. Rasmussen encephalitis, another infrequent condition, is distinguished by unilateral hemisphere atrophy, lacking calvarial alterations, and presenting with localized epilepsy and cognitive impairments [19].

The therapeutic objective of DDMS is to effectively manage convulsions, hemiplegia, hemiparesis, and learning disabilities. A more favorable prognosis is anticipated if hemiparesis manifests after two years without prolonged or recurrent seizures. In cases where children exhibit hemiplegia, hemispherectomy emerges as a potential intervention, boasting a success rate of 85%. However, for late-onset presentations, a comprehensive approach involving antiepileptic medications, along with physiotherapy, speech therapy, and occupational therapy, proves instrumental in the management of seizures [20].

## Conclusions

DDMS is a rare seizure illness that can be distinguished from other seizure disorders such as Rasmussen encephalitis, Fishman syndrome, Sturge-Weber syndrome, and LNSS. When assessing cerebral hemiatrophy and changes in the structure of the bones, CT and MRI are the preferred methods to use. It is critical to diagnose the condition at its earliest. The goals of treatment should be domiciliary physiotherapy and the best possible control of seizures.
